# Benralizumab: Bringing winds of change to eosinophil-associated diseases^☆^

**DOI:** 10.1016/j.waojou.2025.101153

**Published:** 2025-12-08

**Authors:** Stephanie Korn, Eugene R. Bleecker, Arnaud Bourdin, Christopher McCrae, Maria L. Jison, Andrew Menzies-Gow

**Affiliations:** a Thoraxklinik Heidelberg, Heidelberg, Germany; b IKF Pneumologie GmbH & Co. KG, Mainz, Germany; c Department of Medicine, Mayo Clinic, Scottsdale, AZ, USA; d Université de Montpellier, CHU Montpellier, PhyMedExp, INSERM, CNRS, Montpellier, France; e Translational Science & Experimental Medicine, Research & Early Development, Respiratory & Immunology, BioPharmaceuticals R&D, AstraZeneca, Gaithersburg, MD, USA; f Late R&I Clinical Development, BioPharmaceuticals R&D, AstraZeneca, Gaithersburg, MD, USA; g BioPharmaceuticals Medical, Respiratory and Immunology, AstraZeneca, Cambridge, UK

**Keywords:** Asthma, Benralizumab, Efficacy, Eosinophils, Safety

## Abstract

The majority of patients with severe asthma have an eosinophilic phenotype. Interleukin-5 (IL-5) plays a key role in the pathophysiology of severe eosinophilic asthma (SEA) through its effects on eosinophil maturation, survival, and recruitment to the airways. Benralizumab is an anti-IL-5 receptor α (IL-5Rα) monoclonal antibody that binds to IL-5R on eosinophils. Binding of benralizumab to IL-5R blocks IL-5 binding and leads to eosinophil cell death via natural killer cell-mediated antibody-dependent cellular cytotoxicity, macrophage-mediated antibody-dependent cellular phagocytosis/efferocytosis, and tumour necrosis factor receptor 1-mediated apoptosis; the result is the removal of eosinophils from blood and tissue. Benralizumab was approved for the treatment of SEA in 2017 based on the WINDWARD clinical trial programme, which included the pivotal phase 3 trials SIROCCO and CALIMA. Subsequent clinical studies, as well as real-world evidence studies, have reinforced the efficacy of benralizumab for the treatment of SEA and provided evidence about its safety and tolerability profile, including in children. Clinical data have also demonstrated that reduction of background medications, such as oral and inhaled corticosteroids, is possible in patients with SEA controlled with benralizumab. Benralizumab has been investigated for the treatment of other eosinophil-associated diseases, including a phase 3 study for eosinophilic granulomatosis with polyangiitis (EGPA) in which benralizumab was non-inferior to the anti-IL-5 monoclonal antibody mepolizumab: as a result, benralizumab was recently approved for the treatment of EGPA. Phase 3 studies are ongoing with benralizumab for the treatment of hypereosinophilic syndrome and chronic obstructive pulmonary disease.

## Graphical abstract

**Figure:**
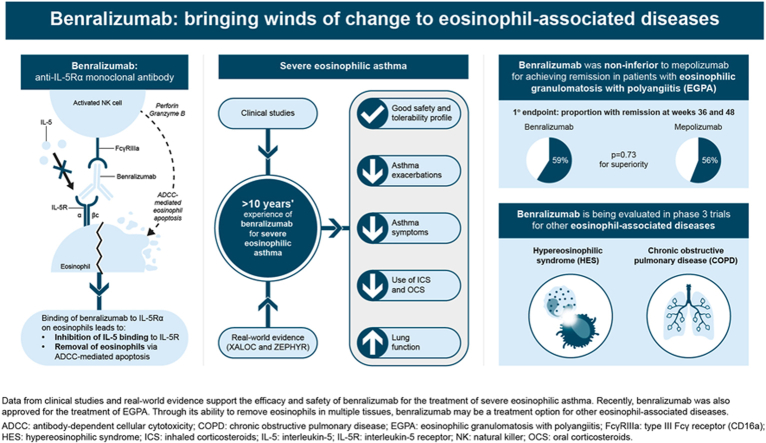

## Introduction

Asthma is a chronic inflammatory disease of the airways affecting ∼300 million people worldwide.^1^ Up to 10% patients with asthma have severe disease, defined as asthma that cannot be controlled with a high-dose inhaled corticosteroid (ICS) plus long-acting beta agonist (LABA) or that gets worse when the dose is reduced.^2^^,^^3^ Asthma exacerbations place a substantial burden on patients and may be life-threatening.^2^

Eosinophils contribute to inflammation and airway remodelling in asthma, leading to progressive airway damage.^4^ Increased blood and sputum eosinophils correlate with worse outcomes, including more frequent severe exacerbations, worse asthma control, and impaired lung function.^5^, ^6^, ^7^, ^8^, ^9^ Eosinophil-associated airway inflammation is a key component of severe eosinophilic asthma (SEA); ∼80% patients with severe asthma have SEA.^10^ Patients with severe asthma and blood eosinophils (bEOS) ≥300 cells/μL, or variable counts, experience greater exacerbation rates than those with bEOS <300 cells/μL.^11^

The advent of biologic therapies for patients with severe asthma that is inadequately controlled by high-dose ICS/LABA has led to substantial changes to Global Initiative for Asthma (GINA) guidelines, including a shift away from oral corticosteroids (OCS).^2^ Biologic therapies for severe asthma include: anti-immunoglobulin (Ig)E (omalizumab); anti-interleukin (IL)-5 (mepolizumab and reslizumab); anti-IL-5 receptor α (IL-5Rα; benralizumab); and anti-IL-4Rα (dupilumab) and anti-thymic stromal lymphopoietin (tezepelumab).^12^ In patients with SEA, benralizumab is administered by subcutaneous injection every 4 weeks (Q4W) for the first 3 doses and once every 8 weeks (Q8W) thereafter.^13^ Administration of other biologics for the treatment of asthma is Q4W or more frequently.^14^, ^15^, ^16^, ^17^

The aim of this review is to provide an up-to-date overview of the anti-IL-5/Rα monoclonal antibody (mAb), benralizumab, in the treatment of eosinophil-associated diseases, with a focus on SEA.

## Selection of sources

PubMed was used to source mechanistic and clinical data for benralizumab using the search term “benralizumab”. Publications considered to report key mechanistic and clinical data for benralizumab in SEA and other diseases were selected.

## Role of IL-5 in SEA

Eosinophilic airway inflammation in type 2 asthma is driven by the release of alarmins thymic stromal lymphopoietin, IL-25 and IL-33, in the bronchial epithelium, driven by external stimuli such as inhaled allergens and airborne pollutants.^18^ The presence of alarmins activates an inflammatory cascade, inducing the release of type 2 cytokines IL-4, IL-5 and IL-13 to amplify type 2 inflammation.^18^ Consistent with the importance of these mediators in asthma pathophysiology, approved biologic therapies for severe asthma target IgE, IL-4, IL-5, or thymic stromal lymphopoietin.^12^

IL-5 is a type 2 cytokine^18^ released by cells in the inflammatory cascade upon activation by external stimuli, such as inhaled allergens and airborne pollutants.^19^ IL-5 is the main cytokine responsible for eosinophil maturation, survival and recruitment to the airways; it exerts its effects by binding to IL-5R, which is highly expressed by eosinophils.^19^ Although the importance of IL-5 and eosinophils in asthma was initially uncertain, studies using anti-IL-5 mAbs confirmed their key roles in it;^20^^,^^21^ as such, IL-5 was identified as a suitable therapeutic target for SEA.^19^

## Benralizumab mechanism of action

**Fig. 1:**
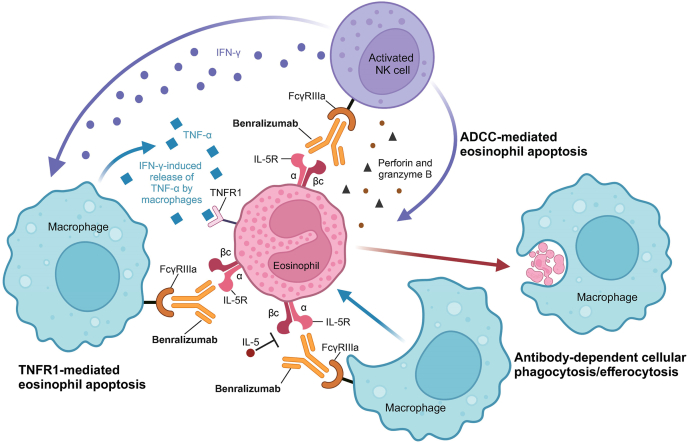
Mechanism of action of benralizumab leading to removal of eosinophils from blood and tissue. Benralizumab binding to IL-5R⍺ on eosinophils directly inhibits the binding of IL-5 to IL-5R. In addition, benralizumab recruits NK cells via binding of its Fc domain to FcγRIIIa, leading to ADCC-mediated apoptosis of eosinophils. Benralizumab recruits macrophages in a similar manner, resulting in antibody-dependent cellular phagocytosis/efferocytosis. Lastly, IFN-γ released by NK cells stimulates the release of TNF-⍺ by macrophages, leading to TNF-⍺/TNFR1-mediated apoptosis of eosinophils. Together, these mechanisms contribute to the removal of eosinophils from blood and tissue. Figure was created in BioRender. Ramos, J. (2025) https://BioRender.com/7gfwnff. ADCC, antibody-dependent cellular cytotoxicity; FcγRIIIa, type III Fcγ receptor (CD16a); IFN-γ: interferon gamma, IL-5, interleukin-5; IL-5R, interleukin-5 receptor; NK, natural killer; TNF-α, tumour necrosis factor alpha; TNFR1, tumour necrosis factor receptor 1

Benralizumab is a humanised afucosylated IgG1 anti-IL-5/Rα mAb.^22^ IL-5Rα is predominantly expressed on eosinophils and basophils.^22^ *In vitro* data have shown that benralizumab binds to IL-5Rα close to the IL-5 binding site and inhibits IL-5-dependent proliferation of cells transfected with recombinant human IL-5Rαβ.^22^ The lack of fucosylation of the oligosaccharide core of IgG1 in benralizumab results in increased affinity for human FcγRIIIa, the key receptor for Fcγ expressed on natural killer (NK) cells, neutrophils and macrophages. Thus, in addition to inhibiting IL-5 binding to IL-5Rα, benralizumab recruits NK cells leading to eosinophil apoptosis via antibody-dependent cellular cytotoxicity (ADCC) (Fig. 1).^22^^,^^23^ Based on *in vitro* data, in a process mediated by interaction between benralizumab and FcγRIIIa on macrophages, benralizumab can also induce antibody-dependent cellular phagocytosis/efferocytosis of eosinophils (Fig. 1).^24^ In addition, activated NK cells release interferon-γ, which stimulates macrophages to release tumour necrosis factor-(TNF)α; subsequent activation of TNF-receptor 1 (TNFR1) on eosinophils results in TNFR1-mediated eosinophil apoptosis (Fig. 1).^24^ Together, these mechanisms contribute to removal of eosinophils from blood and tissue, bone marrow, airway mucosa and sputum.^23^^,^^25^, ^26^, ^27^, ^28^, ^29^, ^30^ Benralizumab has been shown to reduce other cell types expressing IL-5Rα, including basophils (which contribute to an asthma endotype characterised by peripheral blood basophilia), IL-5Rα-positive type 2 innate lymphoid cells, and eosinophil lineage-committed progenitor cells.^23^^,^^29^^,^^31^^,^^32^

In patients with asthma, benralizumab decreased serum levels of eosinophil-derived neurotoxin and eosinophil cationic protein, but increased IL-5, eotaxin-1 and eotaxin-2.^29^^,^^33^, ^34^, ^35^ Similarly, benralizumab increased eotaxin-1 and eotaxin-2 in patients with chronic obstructive pulmonary disease (COPD).^35^ In patients with SEA and high baseline fractional exhaled nitric oxide (FeNO; biomarker of type 2 inflammation), benralizumab was associated with decreased FeNO.^36^ In both patients with asthma and patients with COPD, benralizumab treatment reduced expression of several eosinophil- or basophil-related genes.^35^

In severe asthma, chronic eosinophilic activity may promote mucus plugging associated with chronic airflow obstruction.^37^^,^^38^ In prednisone-dependent patients with asthma and sputum eosinophils ≥3%, magnetic resonance imaging (MRI) ventilation-defect-percentage (VDP; measure of asthma airway dysfunction) was improved pre- and post-bronchodilator after treatment with benralizumab, mepolizumab, reslizumab, or dupilumab.^39^ In addition, treatment with benralizumab decreased the number of mucus plugs.^40^ Finally, a single dose of benralizumab improved both ^129^Xe MRI VDP and asthma control (Asthma Control Questionnaire-6 [ACQ-6] score) at Day 28 in patients with poorly controlled asthma and a high degree of mucus plugging, but not in those with a lower degree of mucus plugging.^41^ The early MRI VDP response persisted, and asthma control continued to improve after further benralizumab treatment up to 2.5 years, along with improvement in computed tomography (CT) mucus score.^42^

## Benralizumab for treatment of SEA

Since its first approval for SEA in the United States in 2017, benralizumab is now approved in >80 countries including, most recently, China.^43^ In Europe, benralizumab is indicated for add-on maintenance treatment in adults with SEA.^44^ Benralizumab is administered as a single 30 mg subcutaneous (SC) injection Q4W for the first three doses, followed by Q8W.^44^ The results of studies that assessed benralizumab for the treatment of SEA are detailed below.

### Phase 1 and 2 studies

Phase 1 studies in asthma showed benralizumab induced rapid and persistent removal of eosinophils from blood, airway mucosa, sputum and bone marrow.^23^^,^^34^^,^^43^^,^^45^ In phase 2 studies in asthma assessing a range of benralizumab doses (2–200 mg SC), benralizumab resulted in near complete and persistent removal of bEOS from Day 1, and reduced asthma exacerbations and improved lung function compared with placebo.^34^^,^^46^^,^^47^ Benralizumab had an acceptable safety profile during phase 1 and 2 studies, supporting evaluation of benralizumab 30 mg SC in phase 3 studies.^23^^,^^33^^,^^43^^,^^45^, ^46^, ^47^

### Phase 3 and 4 studies

Several phase 3 and 4 studies have evaluated benralizumab in patients with severe asthma; their study aims, designs, patient populations, treatment arms and main results are summarised in Table 1 and key studies are discussed below. Central to approval of benralizumab for SEA was the WINDWARD programme, which included six phase 3 studies: SIROCCO, CALIMA, BORA, ZONDA, GREGALE, and BISE (BISE is excluded from Table 1 as it was conducted in patients with mild-to-moderate asthma to meet US Food and Drug Administration regulatory requirements).^48^, ^49^, ^50^, ^51^, ^52^, ^53^, ^54^

**Table 1:** Phase 3 and 4 clinical studies of benralizumab in patients with severe asthma.

Study name	Study aim	Study design	Patient population	Treatment arms	Key results
**SIROCCO**^48^	***EFFICACY & SAFETY*** Assess efficacy and safety of benralizumab in patients with severe asthma inadequately controlled with high-dose ICS plus LABA	Phase 3, 48-week, randomised, double-blind, parallel group, placebo-controlled, multicentre	Aged 12–75 years Diagnosis of asthma for ≥1 year ≥2 exacerbations while taking medium or high-dose ICS plus LABA in previous year	Benralizumab 30 mg SC Q4W (n = 399) Benralizumab 30 mg SC Q8W (Q4W for first 3 doses) (n = 398) Placebo SC Q4W (n = 407)	*Patients with baseline bEOS ≥300 cells/μL* Compared with placebo, over 48 weeks benralizumab: **Reduced AER** Q4W rate ratio: 0.55 (95% CI 0.42–0.71; p < 0.0001) Q8W rate ratio: 0.49 (95% CI 0.37–0.64; p < 0.0001) **Improved pre-BD FEV_1_** Q4W LS mean difference: 0.106 L (95% CI 0.016–0.196; p = 0.0215) Q8W LS mean difference: 0.159 L (95% CI 0.068–0.249; p = 0.0006) **Improved asthma symptom score**^a^ Q4W LS mean difference: −0.08 (95% CI –0.27 to 0.12; p = 0.4420) Q8W LS mean difference: −0.25 (95% CI –0.45 to −0.06; p = 0.0118)
**CALIMA**^53^	***EFFICACY & SAFETY*** Assess efficacy and safety of benralizumab in patients with SEA inadequately controlled with high-dose ICS plus LABA	Phase 3, 56-week, randomised, double-blind, parallel group, placebo-controlled, multicentre	Aged 12–75 years Diagnosis of asthma for ≥1 year ≥2 exacerbations while taking medium or high-dose ICS plus LABA in previous year	Benralizumab 30 mg SC Q4W (n = 425) Benralizumab 30 mg SC Q8W (Q4W for first 3 doses) (n = 441) Placebo SC Q4W (n = 440)	*Patients with baseline bEOS count ≥300 cells/μL* Compared with placebo, over 56 weeks benralizumab: **Reduced AER** Q4W rate ratio: 0.64 (95% CI 0.49–0.85; p = 0.0018) Q8W rate ratio: 0.72 (95% CI 0.54–0.95; p = 0.0188) **Improved pre-BD FEV_1_** Q4W LS mean difference: 0.125 L (95% CI 0.037–0.213; p = 0.0054) Q8W LS mean difference: 0.116 L (95% CI 0.028–0.204; p = 0.0102) **Improved asthma symptom score**^a^ Q4W LS mean difference: −0.12 (95% CI –0.32 to 0.07; p = 0.2241) Q8W LS mean difference: −0.23 (95% CI –0.43 to −0.04; p = 0.0186)
**MIRACLE**^60^	***EFFICACY & SAFETY*** Assess efficacy and safety of benralizumab in patients with severe asthma in China, South Korea and the Philippines	Phase 3, 48-week, randomised, double-blind, parallel group, placebo-controlled, multicentre	Aged 12–75 years ≥2 exacerbations in previous year Asthma requiring treatment with medium or high-dose ICS plus LABA	Benralizumab 30 mg SC Q8W (Q4W for first 3 doses) (n = 348) Placebo SC Q8W (Q4W for first 3 doses) (n = 347)	*Patients with baseline bEOS ≥300 cells/μL* Compared with placebo, over 48 weeks benralizumab: **Reduced AER** Rate ratio: 0.26 (95% CI 0.19–0.36; p < 0.0001) **Improved pre-BD FEV_1_** LS mean difference: 0.25 L (95% CI 0.17–0.34; p < 0.0001) **Improved asthma symptom score** LS mean difference: −0.25 (95% CI –0.45 to −0.05; p = 0.0126)
**BORA**^49^^,^^50^	***LONG-TERM SAFETY*** Assess long-term safety and tolerability of benralizumab in patients with severe asthma	Phase 3, randomised, double-blind, parallel group extension study of SIROCCO AND CALIMA (56 weeks for adults; 108 weeks for adolescents) Participants from ZONDA also eligible for enrolment but results from these patients were not part of this analysis	Previously enrolled in SIROCCO or CALIMA	Patients previously assigned benralizumab 30 mg SC Q4W (n = 633) or benralizumab 30 mg SC Q8W (n = 639) remained on this treatment (Q4W/Q4W and Q8W/Q8W groups, respectively) Patients previously assigned to placebo were randomised to receive benralizumab 30 mg SC Q4W (n = 320) or benralizumab 30 mg SC Q8W (Q4W for first 3 doses) (n = 334) (placebo/Q4W and placebo/Q8W groups, respectively)	1-year results(adults and adolescents) Safety and tolerability outcomes similar to those in the SIROCCO and CALIMA trials No new safety signals **AER** *Patients with baseline bEOS ≥300 cells/μL* Q4W/Q4W: 0.48 (95% CI 0.42–0.56) Placebo/Q4W: 0.53 (95% CI 0.43–0.65) Q8W/Q8W: 0.46 (95% CI 0.39–0.53) Placebo/Q8W: 0.57 (95% CI 0.47–0.68) **Pre-BD FEV_1_ mean change from baseline to Week 48** *Patients with baseline bEOS ≥300 cells/μL* Q4W/Q4W: 0.011 L (95% CI –0.047 to 0.025) Placebo/Q4W: 0.129 L (95% CI 0.057–0.200) Q8W/Q8W: 0.053 L (95% CI 0.015–0.092) Placebo/Q8W: 0.125 L (95% CI 0.065–0.186) 2-year results(86 adolescents only) Both benralizumab Q4W and Q8W regimens were well tolerated with no new safety signals **AER** *Patients with baseline bEOS ≥300 cells/μL* Q4W/Q4W: 0.56 Placebo/Q4W: 0.48 Q8W/Q8W: 0.19 Placebo/Q8W: 0.64 **Pre-BD FEV_1_ mean change from BORA baseline to Week 108** *Patients with baseline bEOS ≥300 cells/μL* Q4W/Q4W: 205 mL Placebo/Q4W: 189 mL Q8W/Q8W: 578 mL Placebo/Q8W: 413 mL
**MELTEMI**^66^	***LONG-TERM SAFETY*** Assess long-term safety and tolerability of benralizumab for up to 5 years in patients with severe asthma	Phase 3, open-label, parallel group, extension safety study of BORA, up to 5 years	Aged 18–75 years Previously enrolled in SIROCCO, CALIMA or ZONDA and then subsequently enrolled in BORA Had received benralizumab treatment for 16–40 weeks in the BORA extension study	Patients previously assigned in predecessor study and BORA to benralizumab 30 mg SC Q4W (n = 150) or benralizumab 30 mg SC Q8W (n = 159) remained on this treatment (Q4W/Q4W and Q8W/Q8W groups, respectively) Patients previously assigned to placebo in predecessor study and then benralizumab 30 mg SC Q4W (n = 70) or benralizumab 30 mg SC Q8W (n = 67) in BORA remained on this treatment (placebo/Q4W and placebo/Q8W groups, respectively)	In an integrated analysis of BORA and MELTEMI, event rates of AEs and SAEs were consistent over time Serious infection, hypersensitivity and malignancy events rates were similar over time No new safety signals **AER** (Extension Year 4) *Patients with baseline bEOS ≥300 cells/μL* Q4W/Q4W: 0.4 Placebo/Q4W: 0.4 Q8W/Q8W: 0.2 Placebo/Q8W: 0.2
**ALIZE**^73^	***SAFETY*** Evaluate the efficacy, safety and immunogenicity of benralizumab on the humoral immune response after seasonal influenza virus vaccination in patients with moderate-to-severe asthma	Phase 3b, 12-week, randomised, double-blind, parallel group, placebo-controlled, multicentre	Aged 12–21 years Uncontrolled asthma Current treatment with ICS plus LABA	Benralizumab 30 mg SC (Weeks 0, 4 and 8) (n = 51) Placebo (Weeks 0, 4 and 8) (n = 52) Tetravalent influenza vaccination at Week 8	Following tetravalent influenza vaccination at Week 8, there were no differences in HAI and MN antibody responses at Week 12 between groups **HAI GMFR from Week 8 to Week 12 for all influenza strains tested** Placebo: 3.4–3.9 Benralizumab Q4W: 3.3–4.2 **MN GMFR from Week 8 to Week 12 for all influenza strains tested** Placebo: 3.2–4.4 Benralizumab Q4W: 2.8–5.1
**TATE**^72^	***PAEDIATRIC POPULATION*** Assess the PK/PD and safety of benralizumab treatment in children with SEA	Phase 3, 48-week, open-label, parallel group, multicentre	Aged 6–11 years for US and Japan (plus aged 12–14 in Japan) Diagnosis of SEA for ≥12 months and bEOS ≥150 cells/μL Treatment with medium-to-high dose ICS in previous 12 months ≥2 exacerbations requiring systemic corticosteroids and/or hospitalisation despite ICS in the previous 12 months	*Weight <*35 kg*:* Benralizumab 10 mg SC on day 0 and weeks 4, 8, 16, 24, 32 and 40 (n = 15) *Weight ≥*35 kg *and Japanese participants aged 12–14 years:* Benralizumab 30 mg SC on day 0 and weeks 4, 8, 16, 24, 32 and 40 (n = 13)	Near complete removal of bEOS for both benralizumab doses Safety profile consistent with previous studies in adolescents/adults No new safety signals Numerical improvements in mean FEV_1_ and ACQ-IA scores **Reduction in exacerbations per patient-treatment year during study** Benralizumab 10 mg: 46% Benralizumab 30 mg: 54%
**SOLANA**^59^	***EFFICACY*** Characterise onset and maintenance of effect of benralizumab in patients with SEA	Phase 3b, 12-week, randomised, double-blind, parallel group, placebo-controlled, multicentre	Aged 18–75 years bEOS ≥300 cells/μL Severe asthma requiring treatment with ICS plus LABA ≥2 exacerbations in previous 12 months	Benralizumab 30 mg SC Q4W for 3 doses (n = 118) Placebo SC Q4W for 3 doses (n = 115)	Compared with placebo, over 12 weeks benralizumab: **Improved pre-BD FEV_1_** LS mean difference: 57 mL (95% CI –22 to 135; p = 0.16) **Improved ACQ-6 score** LS mean difference: −0.395 (95% CI –0.603 to −0.188; p = 0.0002) **Improved SGRQ score** LS mean difference: −7.257 (95% CI –11.133 to −3.380; p = 0.0003)
**ANDHI**^57^^,^^58^	***EFFICACY*** Assess the efficacy of benralizumab in patients with SEA, including onset/impact on health-related quality of life, lung function, exacerbation rate and nasal polyps symptoms	Phase 3b, 24-week, randomised, double-blind, parallel group, placebo-controlled, multicentre	Aged 18–75 years bEOS ≥150 cells/μL Severe asthma requiring treatment with high-dose ICS plus LABA ≥2 exacerbations in previous 12 months	Benralizumab 30 mg SC Q8W (Q4W for first 3 doses) (n = 427) Placebo SC Q8W (n = 229)	Compared with placebo, over 24 weeks benralizumab: **Reduced AER** 49% reduction Rate ratio: 0.51 (95% CI 0.39–0.65; p < 0.0001) **Improved FEV_1_** LS mean difference: 160 mL (95% CI 90–230; p < 0.0001) **Improved ACQ-6 score** LS mean difference: −0.46 (95% CI –0.65 to −0.27; p < 0.0001) **Improved SGRQ score** LS mean difference: −8.11 (95% CI –11.41 to −4.82; p < 0.0001) Enhanced effect of benralizumab on asthma outcomes in subgroup with nasal polyps Compared with placebo, over 24 weeks benralizumab: **Reduced AER** 69% reduction (95% CI 50–81; p < 0.0001) **Improved pre-BD FEV_1_** LS mean difference: 0.32 L (95% CI 0.06–0.47; p < 0.0001) **Improved ACQ-6 score** LS mean difference: −0.88 (95% CI –1.25 to −0.51; p < 0.0001) **Improved SGRQ score** LS mean difference: −16.70 (95% CI –23.3 to −10.2; p < 0.0001) Benralizumab improved nasal polyps symptoms (SNOT-22 score) versus placebo LS mean difference at Week 24: −10.44 (95% CI –19.02 to −1.86; p = 0.0176)
**ANDHI in Practice**^62^	***BACKGROUND MEDICATION REDUCTION*** Assess the potential to reduce standard of care asthma medications while maintaining asthma control with benralizumab	56-week, open-label, Single-arm extension of ANDHI study	Previously enrolled in ANDHI	Benralizumab 30 mg SC Q8W (Q4W for first 3 doses) (n = 503)	By EOT in non-OCS-dependent patients 53% patients reduced ≥1 background medications; increased to 73% in those with asthma control at EOT 39% patients taking LAMA, 34% patients taking LTRA and 12% patients taking LABA at baseline eliminated these medications 42% and 19% patients achieved ≥1 and ≥ 2 adapted GINA step reductions from baseline to EOT, respectively By EOT in OCS-dependent patients 51% patients eliminated OCS use by EOT
**ZONDA**^54^	***OCS REDUCTION*** Assess ability of benralizumab to reduce OCS use while maintaining asthma control in patients with severe asthma inadequately controlled by high-dose ICS plus LABA and chronic OCS	Phase 3, 28-week, randomised, double-blind, parallel group, placebo-controlled, multicentre	Aged 18–75 years bEOS ≥150 cells/μL Asthma treated with chronic OCS therapy for ≥6 months directly preceding enrolment along with use of ICS plus LABA (high-dose ICS for ≥6 months preceding enrolment; medium- to high-dose ICS for ≥12 months preceding enrolment) ≥1 exacerbation in previous 12 months	Benralizumab 30 mg SC Q4W (n = 72) Benralizumab 30 mg SC Q8W (Q4W for first 3 doses) (n = 73) Placebo SC Q4W (n = 75)	**Median reduction in OCS dose from baseline to Week 28 (range)**^b^ Placebo: 25.0% (–150 to 100) Benralizumab Q4W: 75.0% (–100 to 100); p < 0.001 versus placebo Benralizumab Q8W: 75.0% (–50 to 100); p < 0.001 versus placebo **Proportion with ≥90% reduction in OCS dose from baseline to Week 28** Placebo: 12% Benralizumab Q4W: 33% Benralizumab Q8W: 37%
**PONENTE**^63^	***OCS REDUCTION*** Assess the efficacy and safety of tapering OCS use during benralizumab treatment in patients with SEA	Phase 3, open-label, multicentre	Aged ≥18 years bEOS ≥150 cells/μL (or ≥300 cells/μL in previous year) Asthma treated with high-dose ICS plus LABA for ≥6 months and OCS for ≥3 months before enrolment	Benralizumab 30 mg SC Q8W (Q4W for first 3 doses) (n = 598) *OCS tapering* Rate and magnitude of OCS dose reduction from Week 4 dependent on baseline OCS dose, asthma control and adrenal insufficiency status	63% (95% CI 59–67) patients eliminated OCS use for ≥4 weeks 82% (95% CI 79–85) patients eliminated OCS use or achieved daily OCS dose of ≤5 mg for ≥4 weeks, if further reduction was not possible due to adrenal insufficiency 75% patients had no asthma exacerbations during OCS tapering
**SHAMAL**^64^	***ICS REDUCTION*** Assess potential for reduction of ICS maintenance dose during benralizumab treatment in patients with SEA	Phase 4, 48-week, randomised, open-label, Active-controlled, multicentre 32-week reduction period followed by a 16-week maintenance period	Aged ≥18 years SEA controlled with high-dose ICS plus LABA Received ≥3 doses of benralizumab before screening	*Reduction arm:* Benralizumab 30 mg SC Q8W + high-dose ICS/LABA tapered to medium dose, low dose and as-needed dose (n = 125) *Reference arm:* Benralizumab 30 mg SC Q8W + high-dose ICS/LABA + SABA as needed (n = 43)	**Proportion with ICS/LABA dose reduction by end of reduction period (Week 32)** Any dose reduction: 92% Medium dose: 15% Low dose:17% As-needed AIR: 61% **Change in ICS/LABA dose from Week 32 to Week 48** No change: 96% Decreased: 2% Increased: 3% 91% patients in the reduction group had no asthma exacerbations during tapering
**GRECO**^132^	***ADMINISTRATION*** Assess usability of autoinjector for benralizumab administration for patients with severe asthma	Phase 3, 28-week, non-randomised, open-label, multicentre	Aged 18–75 years Severe, uncontrolled asthma Current treatment with ICS plus LABA	Benralizumab 30 mg Q4W (5 doses in total) (n = 121) Administered using a single-use autoinjector device: Week 0 – administered by HCP Week 4 – administered by HCP or patient/caregiver under supervision Week 8 – administered by patient/caregiver under supervision Week 12 and 16 – administered by patient/caregiver at home	At-home administration of benralizumab via autoinjector was successful for 97% patients at Week 12 and 97% at Week 16 ACQ-6 score improved from baseline to Week 20 Removal of bEOS by Week 20 and maintained to Week 28 No new safety signals
**GREGALE**^52^	***ADMINISTRATION*** Assess usability of accessorised pre-filled syringe for benralizumab administration for patients with severe asthma	Phase 3, 28-week, non-randomised, open-label, multicentre	Aged 18–75 years Severe, uncontrolled asthma Current treatment with ICS plus LABA	Benralizumab 30 mg Q4W (5 doses in total) (n = 116) Administered using an accessorised pre-filled syringe: Week 0 – administered by HCP Week 4 – administered by HCP or patient/caregiver under supervision Week 8 – administered by patient/caregiver under supervision Week 12 and 16 – administered by patient/caregiver at home	At-home administration of benralizumab via accessorised pre-filled syringe was successful for 98% patients at Week 12 and 99% at Week 16 ACQ-6 score improved from baseline to Week 20 Removal of bEOS by Week 20 and maintained to Week 28 No new safety signals
**BenRex**^61^^,^^133^	***EXACERBATIONS*** Characterise asthma exacerbations that occur during treatment with benralizumab in patients with SEA	Phase 4, 56–80-week, open-label, prospective, multicentre	Aged 18–80 years SEA treated with high-dose ICS plus >1 additional drug for asthma	Benralizumab 30 mg SC	During exacerbations on benralizumab over a 56–80-week period, eosinophils remained suppressed in the blood and airways of patients FeNO remained elevated Infective features such as sputum neutrophilia and a rise in C-reactive protein occurred
**CHINOOK**^76^	***AIRWAY REMODELLING*** Assess effect of benralizumab on lung structure and function in patients with SEA	Phase 4, 48-week, randomised, double-blind, parallel group, placebo-controlled, multicentre	Aged 18–70 years SEA treated with high-dose ICS plus LABA	Benralizumab 30 mg SC Q8W (Q4W for first 3 doses) Placebo SC Q8W (Q4W for first 3 doses)	Study in progress Primary outcomes that will be assessed (from baseline to Week 48): Change in eosinophil count in submucosa Change in wall area percentage in airway as the overall median for airway generations 3 and 4 combined determined by CT imaging
**BURAN**^77^	***AIRWAY DYNAMICS*** Assess effect of benralizumab on airway geometry and dynamics in patients with SEA	Phase 4, 13-week, single arm, uncontrolled, open-label, multicentre	Aged 18–70 years SEA inadequately controlled by ICS plus LABA ( ± OCS or other asthma medications) ≥2 exacerbations in previous year	Benralizumab 30 mg SC (Weeks 0, 4 and 8)	Study in progress Functional respiratory imaging, a novel technique, will be used to assess lung structure and function at weeks 0 and 13
**BRISOTE**^78^	***EFFICACY & SAFETY*** Assess efficacy and safety of benralizumab as an add-on therapy in patients with eosinophilic asthma that is uncontrolled on medium-dose ICS LABA	Phase 3b, 48-week, randomised, double-blind, parallel group, active-controlled, multicentre	Aged 12–75 years Diagnosis of asthma requiring treatment with at least medium-dose ICS and a LABA, for ≥1 year Treatment with medium-dose ICS and LABA for ≥3 months with or without asthma controllers (excluding OCS) ≥2 exacerbations in previous year	Medium-dose ICS-LABA plus benralizumab 30 mg SC Q8W (Q4W for first 3 doses) High-dose ICS-LABA plus placebo SC Q8W (Q4W for first 3 doses)	Study in progress Primary outcome that will be assessed is annualised asthma exacerbation rate (from baseline to Week 48)

ACQ-6, Asthma Control Questionnaire 6; ACQ-IA, Asthma Control Questionnaire-Interviewer administered; AE, adverse event; AER, annual exacerbation rate; AIR, anti-inflammatory reliever; bEOS, blood eosinophils; CI, confidence interval; CT, computed tomography; EOT, end of treatment; FEV_1_, forced expiratory volume in 1 s; GINA, Global Initiative for Asthma; GMFR, geometric mean fold rise; HAI, haemagglutination inhibition; HCP, healthcare professional; ICS, inhaled corticosteroids; LABA, long-acting beta agonist; LAMA, long-acting muscarinic antagonist; LS, least-squares; LTRA, leukotriene receptor antagonist; MN, microneutralisation; OCS, oral corticosteroid; PD, pharmacodynamics; PK, pharmacokinetics; pre-BD, pre-bronchodilator; Q4W, every 4 weeks; Q8W, every 8 weeks; SABA, short-acting beta agonist; SAE, serious adverse event; SC, subcutaneous; SEA, severe eosinophilic asthma; SGRQ, St. George's Respiratory Questionnaire; SNOT-22, Sino-Nasal Outcome Test-22.

a Asthma symptom score was a composite of daytime and night-time symptoms (scored 0–6; decrease indicates improvement).

b A negative value indicates an increase in final OCS dose from baseline

**Fig. 2:**
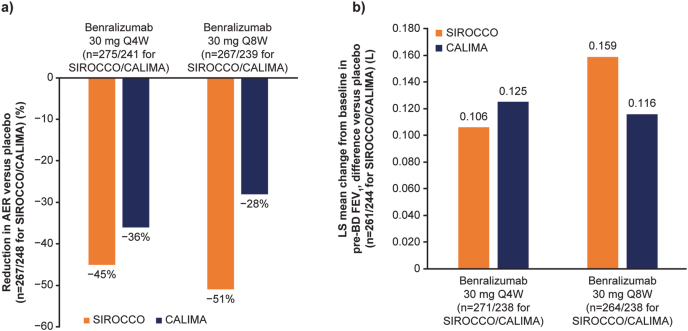
Effect of benralizumab on AER and lung function in SIROCCO and CALIMA in patients with bEOS ≥300 cells/μL.^48^^,^^53^ a) AER reduction with benralizumab versus placebo. b) LS mean change from baseline in pre-BD FEV_1_, difference versus placebo. Timeframes for SIROCCO and CALIMA data are 48 and 56 weeks, respectively. AER, annual exacerbation rate; bEOS, blood eosinophils; FEV1, forced expiratory volume in 1 second; LS, least-squares; BD, bronchodilator; Q4W, every 4 weeks; Q8W, every 8 weeks

Two pivotal randomised, double-blind, placebo-controlled phase 3 studies, SIROCCO and CALIMA, were conducted in patients with severe asthma; their aim was to assess efficacy and safety of benralizumab 30 mg Q4W or Q8W (Q4W for the first 3 dose) versus placebo over 48 and 56 weeks, respectively.^48^^,^^53^ In SIROCCO and CALIMA, compared with placebo, benralizumab Q8W significantly reduced the annual exacerbation rate (AER) by 51% (p < 0.0001) and 28% (p = 0.0188), respectively, in patients with baseline bEOS ≥300 cells/μL (Table 1, Fig. 2A).^48^^,^^53^ In addition, benralizumab significantly improved lung function (forced expiratory volume in 1 second [FEV_1_]; Table 1, Fig. 2B). Asthma symptom score was significantly improved for benralizumab Q8W dosing only (Table 1).^48^^,^^53^ A positive anti-drug antibody response was reported in 13–15% of patients in SIROCCO and CALIMA, but this was not associated with hypersensitivity and did not affect efficacy outcomes.^48^^,^^53^

A pooled analysis of SIROCCO and CALIMA revealed that improved efficacy of benralizumab was associated with baseline OCS use, nasal polyposis, pre-bronchodilator (BD) forced vital capacity (FVC) < 65% of predicted, ≥3 exacerbations in previous year and diagnosis in adulthood for all patients as well as those with bEOS ≥300 cells/μL.^55^ In addition, multivariate cluster analyses found greater reductions in exacerbations in patient clusters with late-onset asthma;^56^ they also showed greater improvements in FEV_1_ and FVC in the cluster with fixed airflow obstruction (FAO).^56^

ANDHI assessed AER, lung function and quality of life versus placebo.^57^ Over 24 weeks, benralizumab 30 mg Q8W reduced AER by 49% (p < 0.0001), and improved FEV_1_, ACQ-6 score and St George's Respiratory Questionnaire (SGRQ) score (Table 1).^57^ In the ANDHI Nasal Polyp substudy of patients with asthma and comorbid nasal polyps (NP), benralizumab had an enhanced benefit on asthma outcomes and improved NP symptoms (Sino-Nasal Outcome Test-22 [SNOT-22] score) (Table 1).^58^

SOLANA characterised onset and maintenance of the effect of benralizumab versus placebo in SEA.^59^ Over 12 weeks, benralizumab 30 mg Q4W increased pre-BD FEV_1_ (p = 0.16) and significant improvements in ACQ-6 and SGRQ scores were observed (Table 1).^59^

Efficacy/safety of benralizumab in patients with severe asthma in China, South Korea and the Philippines was investigated in MIRACLE.^60^ Over 48 weeks in patients with baseline bEOS ≥300 cells/μL, benralizumab 30 mg Q8W reduced AER by 74% (p < 0.0001), and improved pre-BD FEV_1_ and asthma symptom score, versus placebo (Table 1).^60^

BenRex was an open-label, prospective study that characterised asthma exacerbations occurring during benralizumab treatment.^61^ Over 56–80 weeks of treatment, 58% of enrolled patients had an exacerbation, during which eosinophils were suppressed in the blood and airways.^61^

#### Reduction/elimination of background medications

In patients with severe asthma controlled on biologics, the GINA report recommends reducing ICS dose and eliminating OCS where possible to prevent adverse effects on health; however, there is little guidance to support such approaches.^2^ Reduction/elimination of background medications was investigated in several phase 3/4 benralizumab studies.^54^^,^^62^, ^63^, ^64^

**Fig. 3:**
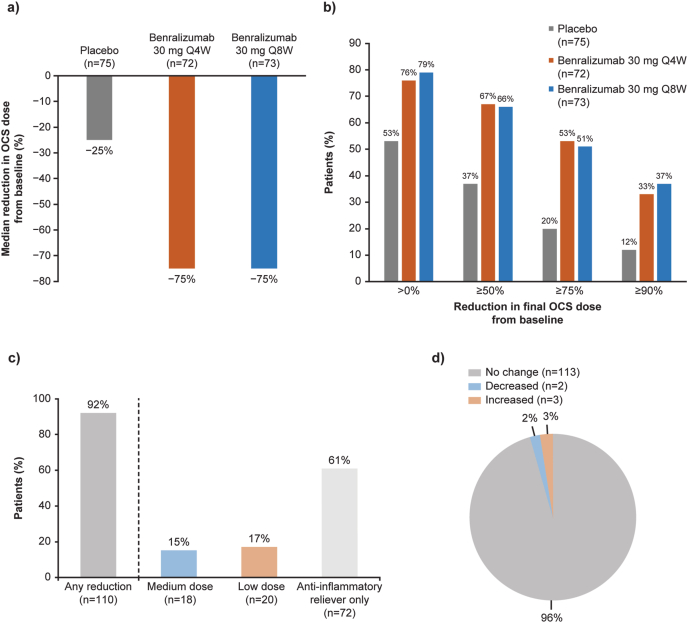
Reduction of OCS dose (ZONDA study) and ICS/LABA dose (SHAMAL study) in patients with SEA controlled on benralizumab.^54^^,^^64^ ZONDA study: a) Median reduction in OCS dose as a percentage of baseline value at Week 28; b) Proportion of patients with different levels of OCS dose reduction from baseline to final dose (Week 28). SHAMAL study: c) Proportion of patients reducing their ICS/LABA dose at the end of the reduction period (Week 32); d) Proportion of patients maintaining their ICS/LABA dose at Week 32 through to Week 48 (end of the maintenance period). Parts c) and d) adapted from Jackson DJ et al. Reduction of daily maintenance inhaled corticosteroids in patients with severe eosinophilic asthma treated with benralizumab (SHAMAL): a randomised, multicentre, open-label, phase 4 study. *Lancet* 2024; 403: 271–281; originally published under CC-BY. ICS, inhaled corticosteroid; LABA, long-acting beta agonist; OCS, oral corticosteroid; Q4W, every 4 weeks; Q8W, every 8 weeks; SEA, severe eosinophilic asthma

The double-blind ZONDA study demonstrated a median 75% reduction in OCS dose from baseline to Week 28 for benralizumab 30 mg Q4W and Q8W versus 25% for placebo (p < 0.001 for both comparisons); OCS dose was reduced by ≥ 90% in 12%, 33% and 37% patients who received placebo, benralizumab Q4W and benralizumab Q8W, respectively (Table 1, Fig. 3A and B).^54^

In the open-label PONENTE study, 63% patients receiving benralizumab 30 mg Q8W eliminated OCS for ≥4 weeks using an individualised OCS tapering algorithm; 82% patients eliminated OCS or achieved daily OCS dose of ≤5 mg for ≥4 weeks if further reduction was not possible due to adrenal insufficiency (Table 1).^63^

The open-label SHAMAL study evaluated ICS/LABA dose reduction in patients with SEA controlled with benralizumab over a 32-week reduction period followed by a 16-week maintenance period.^64^ Overall, 92% patients reduced ICS/LABA dose by Week 32, with 61% to ICS/LABA as-needed anti-inflammatory reliever only; these reductions were maintained to Week 48 in 96% patients (Table 1; Fig. 3C and D).^64^

Finally, in ANDHI in Practice (56-week single arm, open-label extension [OLE] study of ANDHI), 53% patients receiving benralizumab 30 mg Q8W reduced ≥1 background medication by end of treatment (EOT); 39% patients taking long-acting muscarinic antagonists (LAMAs) and 34% patients taking leukotriene receptor antagonists eliminated these medications (Table 1).^62^ In total, 42% and 19% patients achieved ≥1 or ≥2 adapted GINA step reductions from baseline to EOT, respectively.^62^

#### Additional analyses of benralizumab clinical studies

**Fig. 4:**
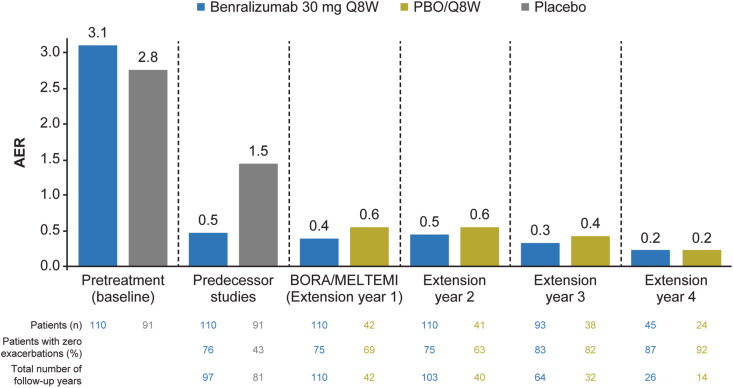
Long-term efficacy of benralizumab: an integrated analysis of patients from SIROCCO, CALIMA and ZONDA who enrolled in the BORA and MELTEMI extension studies.^66^ AER for patients with bEOS ≥300 cells/μL receiving high-dosage ICS at predecessor baseline. Placebo (n = 91) from predecessor studies includes 49 patients in the PBO/Q4W group and 42 patients in the PBO/Q8W group during the extension studies. Adapted from Korn S et al. Integrated safety and efficacy among patients receiving benralizumab for up to 5 years. *J Allergy Clin Immunol Pract* 2021; 9: 4381–4392; originally published under CC-BY. AER, annual exacerbation rate; bEOS, blood eosinophils; ICS, inhaled corticosteroid; PBO, placebo; Q4W, every 4 weeks; Q8W, every 8 weeks

Several additional analyses of clinical studies have further evaluated benralizumab. Data from pivotal 48-week SIROCCO and 56-week CALIMA studies were merged with 56-week data for adults from the BORA phase 3 extension study in a 2-year integrated analysis.^65^ This showed AER reduction and lung function improvement with benralizumab 30 mg Q8W were maintained over 2 years (bEOS ≥300 cells/μL: 2-year integrated AER, 0.56 [95% confidence interval [CI] 0.51–0.62]; mean pre-BD FEV_1_ increase from baseline, 0.343 L at 1 year and 0.364 L at 2 years).^65^ Expanding upon this, an integrated analysis of patients from SIROCCO, CALIMA and ZONDA who subsequently enrolled in the BORA and MELTEMI extension studies showed that AER reduction following treatment with benralizumab 30 mg Q8W was maintained up to 5 years (Fig. 4).^66^

An integrated analysis of ZONDA and the BORA extension study showed that the 75% reduction in OCS dose achieved with benralizumab 30 mg Q8W during the 28-week ZONDA study was maintained during BORA, with a median 67% reduction from baseline at Week 56.^67^

A *post hoc* analysis of pooled data from SIROCCO and CALIMA (benralizumab 30 mg Q8W or placebo; baseline bEOS ≥300 cells/μL) demonstrated similar reductions in AER with benralizumab versus placebo in patients with and without FAO (rate ratio: 0.56 [95% CI 0.44–0.71; p < 0.0001] and 0.58 [95% CI 0.41–0.83; p = 0.0030], respectively).^68^ Compared with placebo, improvement in lung function (pre-BD FEV_1_) from baseline with benralizumab was greater for patients with FAO than without (least-squares [LS] mean difference 0.159 L [95% CI 0.082–0.236; p < 0.0001] and 0.103 L [95% CI –0.008 to 0.215; p = 0.699], respectively).^68^

In another *post hoc* analysis of patients in SIROCCO, CALIMA and ZONDA, clinical asthma remission comprised 4 components: zero exacerbations, ACQ-6 score <1.5 or ≤0.75, pre-BD FEV_1_ increase ≥100 mL from baseline and zero OCS use.^69^ At 6 months, among patients treated with benralizumab 30 mg Q8W, 87% from SIROCCO/CALIMA and 75% from ZONDA achieved ≥2 clinical remission components, while 15% from SIROCCO/CALIMA and 23% from ZONDA achieved all 4.^69^

Another retrospective *post hoc* analysis of pooled data from SIROCCO and CALIMA demonstrated improvements in lung function with benralizumab treatment in patients with eosinophil counts <300 cells/μL and ≥300 cells/μL at baseline.^70^ Regardless of eosinophil counts, pre- and post-BD FEV_1_ improved by 307 mL and 97 mL, respectively, and pre- and post-BD FVC improved by 349 mL and 94 mL, respectively.^70^

#### Safety

After >10 years’ experience with benralizumab in patients with severe asthma, safety data are reassuring, including serious infections and malignancies.^48^, ^49^, ^50^^,^^53^^,^^54^^,^^66^ In SIROCCO, CALIMA and ZONDA, the frequency of adverse events (AEs) and serious AEs (SAEs) was similar to placebo (AEs: 71–75% versus 76–83%; SAEs: 10–12% versus 14–19%, respectively),^48^^,^^53^^,^^54^ and only 2–4% of patients in the benralizumab groups discontinued treatment due to an AE.^48^^,^^53^^,^^54^ Commonly reported AEs included nasopharyngitis, worsening asthma and upper respiratory tract infection (URTI).^48^^,^^53^^,^^54^ In the long-term extension studies BORA and MELTEMI, no new safety signals that may be associated with long-term removal of eosinophils were observed.^49^^,^^50^^,^^66^

A five-year analysis of MELTEMI (OLE study of benralizumab 30 mg Q4W or Q8W for adults who transitioned from BORA extension study) and COLUMBA (OLE study of mepolizumab 100 mg Q4W for patients who transitioned from DREAM study) compared the safety of benralizumab and mepolizumab in patients with SEA.^71^ This analysis in 446 MELTEMI and 347 COLUMBA patients concluded long-term safety and tolerability were generally similar between mepolizumab and benralizumab, despite the mAbs having different mechanisms of action and effects on eosinophil reduction.^71^ In both studies, the most common non-serious AE was viral URTI (benralizumab: Q4W 47.3% and Q8W 46.5%; mepolizumab: 48.7%) and the most common SAE was asthma-related events (benralizumab: Q4W 8.6% and Q8W 8.0%; mepolizumab: 9.5%).^71^

The recent 48-week, open-label TATE study assessed the pharmacokinetics (PK), pharmacodynamics (PD) and safety of benralizumab 10 mg (<35 kg) or 30 mg (≥35 kg) in children with SEA aged 6–11 years in the United States and Japan (plus children aged 12–14 years in Japan) (Table 1).^72^ Both doses of benralizumab resulted in near-complete removal of bEOS.^72^ Furthermore, the PK/PD and safety profiles of benralizumab were consistent with previous studies in adolescents and adults, supporting long-term use of benralizumab in children.^72^ These results led to approval of benralizumab in children aged ≥6 years in some countries.^13^

As eosinophils may contribute to immune system response following vaccination, it is important to understand the effect of eosinophil removal following benralizumab treatment on immune response generation.^73^ The ALIZE study demonstrated that benralizumab 30 mg Q4W did not impair antibody response to influenza virus vaccination in patients with moderate-to-severe asthma, as evidenced by no consistent differences in haemagglutination inhibition and microneutralisation antibody responses at Week 12 with benralizumab versus placebo (Table 1).^73^

Regarding benralizumab use during pregnancy, a case series on benralizumab treatment in four pregnant women with severe asthma reported no indication of harm as well as positive maternal outcomes.^74^ In a case of benralizumab treatment during pregnancy in a woman with hypereosinophilic syndrome (HES) and severe eosinophilic gastrointestinal involvement, no safety signals were observed and the woman delivered a healthy baby.^75^ These studies suggest no adverse effects of eosinophil removal in pregnant women, although more data are needed to inform guidelines around use of biologics during pregnancy.

#### Ongoing phase 4 studies

Ongoing phase 4 studies of benralizumab in SEA include CHINOOK, BURAN, and BRISOTE (Table 1). The double-blind, placebo-controlled CHINOOK study will assess the effect of benralizumab on lung structure and function over 48 weeks, including use of CT imaging to analyse airway remodelling and histopathologic examination of airway biopsies.^76^ The open-label, 13-week BURAN study will utilise functional respiratory imaging to assess the effect of benralizumab on airway dynamics, focusing on the impact on airway remodelling by morphological and histological measures.^77^ Finally, the double-blind BRISOTE study will assess the efficacy and safety of benralizumab in patients with eosinophilic asthma that is uncontrolled on medium-dose ICS plus LABA.^78^

### Real-world evidence

#### Retrospective studies

The XALOC programme was developed to assess effectiveness of benralizumab for SEA in the real-world setting in biologic-naïve and biologic-experienced patients. XALOC-1 comprises five observational, retrospective studies: ANANKE (Italy), BETREAT (Portugal), BPAP (UK), ORBE II (Spain), and VOLTS (Canada).^79^, ^80^, ^81^, ^82^, ^83^ Results were published as an integrated analysis^83^ and individually by country.^79^, ^80^, ^81^, ^82^

Country-level data from BPAP, ANANKE, and ORBE II demonstrated effectiveness of benralizumab in reducing exacerbations and OCS use, regardless of previous treatment with biologics.^79^, ^80^, ^81^, ^82^ In BPAP, at Week 48 there was 81% reduction in AER with benralizumab versus baseline (n = 208), and 53% (67/126) patients requiring OCS at baseline had eliminated OCS use.^79^ One-year follow-up data from ORBE II demonstrated 86% reduction in exacerbations from baseline among 204 patients; furthermore, 53% (28/53) of OCS-dependent patients achieved OCS withdrawal.^81^ Longer-term data from patients treated with benralizumab for 96 weeks in the ANANKE study showed 95% reduction in AER from start of treatment (index date) (n = 154 at index date; n = 113 at 96 weeks) and elimination of OCS use in 60% (18/30) patients.^82^

A *post hoc* analysis of ANANKE evaluated the characteristics and long-term clinical outcomes in biologic-naïve (n = 124) and biologic-experienced (n = 38) patients treated with benralizumab for up to 96 weeks.^84^ Of biologic-experienced patients, those who had switched from mepolizumab (n = 17) presented with the longest SEA duration (median: 4.6 years) and the greatest OCS daily dosage (median: 25 mg prednisone equivalent), while those who had switched from omalizumab (n = 21) had the highest severe AER (1.70).^84^ After 96 weeks of treatment with benralizumab, AER was reduced by 86–96% in all subgroups versus AER at index date.^84^ Additional *post hoc* analyses of ANANKE showed benralizumab is effective at reducing AER, regardless of previous biologic use, and in patients with bEOS 300–450 cells/mm^3^.^85^^,^^86^

In a 48-week integrated analysis of XALOC-1 including >1000 patients with SEA, patients treated with benralizumab had substantial improvements in AER from baseline to Week 48 regardless of prior biologic use (83% and 73% reduction overall and in biologic-experienced patients, respectively).^83^ Furthermore, 47% (130/274) patients using OCS at baseline eliminated OCS by Week 48.^83^

The multi-country, retrospective, observational study RANS evaluated the effectiveness of benralizumab on asthma and NP outcomes in 233 patients with SEA and comorbid NP.^87^ In the 12 months post-benralizumab initiation, clinically meaningful improvements in total NP, SNOT-22, Asthma Control Test and ACQ-6 scores were observed in 49%, 68%, 81%, and 57% patients, respectively.^87^

ZEPHYR 1 was a retrospective cohort study that assessed clinical and economic impact of benralizumab treatment for SEA in the United States (12 months before benralizumab initiation versus 12 months after).^88^ Among 204 patients, treatment with benralizumab reduced AER (55% reduction; p < 0.001), OCS use, healthcare resource utilisation (HCRU), and exacerbation-related costs.^88^

ZEPHYR 2 was another retrospective cohort study in the United States, which utilised a larger database than ZEPHYR 1 and included laboratory data; it assessed the effectiveness of benralizumab in patients with SEA and a variety of clinical profiles.^89^ Among 429 patients with SEA and ≥1 eosinophil laboratory measure in the 12 months before initiating benralizumab, AER was reduced following treatment with benralizumab regardless of baseline bEOS level (12 months before benralizumab initiation versus 12 months after: 52–64% reductions; p < 0.001).^89^ In an analysis of biologic-naïve (n = 1292) and biologic-experienced patients (prior omalizumab, n = 205; prior mepolizumab, n = 144), reductions in AER and exacerbation-related HCRU and costs were observed regardless of previous biologic use up to 24-months after benralizumab initiation.^90^ Finally, an analysis of 1406 patients with asthma and concomitant COPD showed treatment with benralizumab reduced the rate of asthma (40% reduction; p < 0.001) and COPD (51% reduction; p < 0.001) exacerbations (12 months before benralizumab initiation versus 12 months after); reductions were observed regardless of baseline bEOS.^91^

ZEPHYR 3 utilised the claims-based Healthcare Integrated Research Database and built upon data from ZEPHYR 1 and 2.^92^ Treatment with benralizumab (n = 506) led to 40% reduction in the number of patients with exacerbations from baseline (12 months before benralizumab initiation) to follow-up (12 months after benralizumab initiation; p < 0.001). Among patients with available electronic medical record data (n = 123), there were numeric reductions in the proportion of patients experiencing asthma-related symptoms.^92^

#### Prospective studies

XALOC-2 is a prospective study of benralizumab treatment of SEA in Belgium, Canada, Germany, and Switzerland.^93^ An 8-week integrated interim analysis of 413 patients demonstrated a reduction from baseline in LS mean ACQ-6 score of −0.7 (95% CI –0.8 to −0.6) at Week 1 and –1.2 (95% CI –1.3 to −1.1) at Week 8. Improvement in ACQ-6 score at Week 8 was more pronounced in patients with bEOS ≥500 cells/μL (LS mean change −1.4 [95 CI –1.6 to −1.2]) and with chronic rhinosinusitis with NP (CRSwNP; LS mean change −1.5 [95% CI –1.7 to −1.3]).^93^ Improvement in ACQ-6 score continued over 1 year and 42% (111/262) patients achieved clinical remission by Week 56.^94^^,^^95^

Data from the ongoing, prospective, non-interventional cohort study of US specialist-treated patients with severe asthma (CHRONICLE) showed patients treated with benralizumab had reductions in annualised rates of asthma exacerbations (65%, p < 0.001) and asthma-related hospitalisations (45%, p = 0.007) for 6–12 months pre-initiation versus 6–12 months post-initiation (n = 166), and had mean 52-week adherence of 83% (measured as proportion of days covered; n = 387).^96^^,^^97^ An analysis of data from CHRONICLE (2018–2020; n = 1236) and the International Severe Asthma Registry (2015–2020; n = 2295) showed that while omalizumab was the most common first biologic in 2015, this had changed to benralizumab by 2019.^98^

## Studies exploring benralizumab in other eosinophil-associated diseases

Studies that have investigated or are currently investigating benralizumab for the treatment of other diseases in which eosinophils were thought to play a key pathogenic role are detailed below.

### Completed and ongoing studies

#### EGPA

Eosinophilic granulomatosis with polyangiitis (EGPA) is a rare vasculitis commonly characterised by asthma and eosinophilia.^99^ The phase 3, randomised, double-blind, head-to-head, non-inferiority MANDARA study evaluated the efficacy/safety of benralizumab 30 mg Q4W (n = 70) versus mepolizumab 300 mg Q4W (n = 70) in adults with relapsing or refractory EGPA.^99^ Benralizumab was non-inferior to mepolizumab for the primary endpoint of remission at Weeks 36 and 48, with an adjusted proportion of patients with remission of 59% in the benralizumab group versus 56% in the mepolizumab group (difference 3% [95% CI –13 to 18; p = 0.73 for superiority]).^99^ Additionally, 41% and 26% patients achieved complete withdrawal of OCS between Weeks 48 and 52 with benralizumab and mepolizumab, respectively (difference 16% [95% CI 1–31]).^99^ The mean (standard deviation) bEOS level decreased from 306.0 (225.0) cells/μL at baseline to 32.4 (40.8) cells/μL at Week 52 in the benralizumab group compared with a decrease from 384.9 (563.6) cells/μL at baseline to 71.8 (54.4) cells/μL at Week 52 in the mepolizumab group.^99^ An exploratory *post hoc* analysis of MANDARA evaluated whether patients with EGPA could achieve asthma remission.^100^ The asthma remission rate for benralizumab and mepolizumab groups by number of components was: 3-component remission, 33.6% and 22.3%, respectively (difference: 11.25%; 95% CI: −3.19 to 25.70; p = 0.127); 4-component remission, 28.0% and 12.4%, respectively (difference: 15.64%; 95% CI: 2.96–28.32; p = 0.016).^100^

Retrospective observational studies have compared benralizumab with mepolizumab in patients with EGPA. In 1 study, the overall efficacy and safety of benralizumab (n = 88) and mepolizumab (n = 88) treatment were comparable; benralizumab was also reported to provide a deeper peripheral eosinophil reduction, and more patients achieved a complete response at 12 months with benralizumab versus mepolizumab (48.1% versus 32.4%).^101^ Similarly, another observational study of 49 patients with EGPA noted that both benralizumab and mepolizumab could reduce annual exacerbation rates and OCS use over 24 months.^102^ The rate of clinical remission was similar between mepolizumab and benralizumab over 12 months, although there was a non-significant trend towards more patients experiencing clinical remission with benralizumab versus mepolizumab at 24 months (69.2% versus 43.5%, p = 0.0882). Moreover, in addition to depleting bEOS, benralizumab, but not mepolizumab, also reduced basophil counts. Benralizumab is now approved for the treatment of EGPA in some countries.^13^

#### Atopic dermatitis (AD)

AD is a chronic, inflammatory skin condition in which eosinophils have been implicated.^103^ The phase 2, randomised, double-blind HILLIER study investigated efficacy of benralizumab in 194 patients with moderate-to-severe AD. From baseline to Week 16, there was no significant difference between benralizumab 30 mg Q4W and placebo groups in improvement in investigator global assessment score (absolute difference versus placebo: −8.62% [95% CI –17.94 to 0.71; p = 0.080]) and change in eczema area and severity index score (LS mean difference versus placebo: 3.19 [95% CI –0.86 to 7.24; p = 0.121]).^104^

#### Chronic spontaneous urticaria (CSU)

CSU is a common, difficult-to-treat skin disease characterised by hives/angioedema in which eosinophils may play a role alongside mast cells.^105^ The phase 2b, randomised, double-blind ARROYO study demonstrated no clinical benefit of benralizumab 30 mg or 60 mg Q4W versus placebo in 155 patients with CSU who were symptomatic despite H_1_ anti-histamine treatment (LS mean difference for change from baseline to Week 12 in Itch Severity Score over 7 days for benralizumab 30 mg versus placebo: −1.01 [95% CI –3.28 to 1.26; p = 0.3824]; benralizumab 60 mg versus placebo: −1.79 [95% CI –4.09 to 0.50; p = 0.1244]).^105^

#### Eosinophilic esophagitis (EoE)

EoE is a chronic disease characterised by eosinophil infiltration into tissue and symptoms related to esophageal dysfunction.^106^ The phase 3, randomised, double-blind MESSINA study assessed the efficacy/safety of benralizumab 30 mg Q4W (n = 103) for treatment of EoE compared with placebo (n = 107).^107^ Although treatment with benralizumab resulted in near-complete removal of eosinophils in esophageal tissue at Week 24, it did not improve dysphagia symptoms compared with placebo (LS mean difference in change from baseline in Dysphagia Symptom Questionnaire score: 3.0 points [95% CI –1.4 to 7.4; p = 0.18]).^107^ Furthermore, measures of EoE-related epithelial pathological features indicated continued disease activity despite substantial eosinophil reduction in esophageal tissue and the blood.^107^

Given the ability of benralizumab to remove eosinophils, its lack of efficacy in the HILLIER, ARROYO and MESSINA studies highlights that inflammation driven by eosinophils may not be central to the pathophysiology of AD, CSU and EoE.^104^^,^^105^^,^^107^

#### HES

HES is another rare disorder presenting with a range of symptoms and organ system involvement; however, a common feature is elevated eosinophil levels.^108^ The phase 3, randomised, double-blind, placebo-controlled NATRON study evaluated the efficacy/safety of benralizumab in patients with HES.^109^ High level results revealed that benralizumab treatment led to clinically meaningful improvements in time to first worsening or flare versus placebo.^110^ Primary results are expected to be presented in 2025.

#### CRSwNP

Eosinophils are thought to play a role in the pathogenesis of CRSwNP, an inflammatory disease of the upper airways.^30^ OSTRO, a phase 3, randomised, double-blind study in 413 patients with CRSwNP, showed treatment with benralizumab 30 mg Q8W improved NP and nasal blockage scores and also led to near-complete removal of eosinophils from blood and NP tissue compared with placebo.^30^^,^^111^ A second phase 3, randomised, double-blind, placebo-controlled trial (ORCHID) assessing benralizumab in patients with CRSwNP with comorbid asthma did not meet its co-primary endpoints.^112^^,^^113^ Results expected in 2025.

#### Allergic asthma

Asthma triggered by exposure to an allergen (allergic asthma) is often associated with airway inflammation driven by eosinophils.^114^ The efficacy of benralizumab for treatment of allergic asthma was recently investigated in a double-blind, phase 3 study.^115^ Adults with mild allergic asthma who had late asthmatic response (LAR) and increased sputum eosinophils after allergen inhalation challenge (AIC) at screening were randomised to either benralizumab 30 mg (n = 23) or placebo (n = 23) (3 doses Q4W, Weeks 0–8).^115^ At Week 9, sputum eosinophil levels were significantly reduced at 7 h post-AIC in the benralizumab group compared with placebo (LS mean difference: −5.81% [95% CI –10.69 to −0.94; p = 0.021]); however, benralizumab did not reduce allergen-induced LAR at Week 9 compared with placebo in this mild allergic asthma population (LS mean difference for change in FEV_1_: 2.54% [95% CI 3.05–8.12; p = 0.363]).^115^

#### Bronchiectasis

Although bronchiectasis has primarily been thought to be caused by the long-term effects of chronic respiratory infections, the role of inflammation and anti-inflammatory interventions are now being considered.^116^ There is increasing evidence of association between eosinophilia and bronchiectasis severity, suggesting asthma may lead to bronchiectasis.^117^^,^^118^ The phase 3, double-blind, placebo-controlled MAHALE study investigated the efficacy and safety of benralizumab for treatment of non-cystic fibrosis bronchiectasis.^119^ Recruitment was terminated, and results are expected in due course.

#### Acute eosinophilic exacerbations

Eosinophilia is a common feature of acute exacerbations of COPD and asthma.^120^^,^^121^ The phase 2, double-blind ABRA study assessed if a single dose of benralizumab 100 mg SC in combination with a short course of OCS (prednisolone 30 mg once daily, 5 days) (n = 52), or alone (n = 53), is superior to a short course of OCS alone (standard of care) (n = 53) for the treatment of acute eosinophilic exacerbations of COPD or asthma (bEOS ≥300 cells/μL).^122^ Treatment failures (death, hospitalisation and any need for re-treatment) at 90 days were reduced in the pooled benralizumab group compared with OCS alone (odds ratio 0.26; p = 0.0005), indicating that benralizumab may be effective for treatment of acute eosinophilic exacerbations.^122^

#### COPD

Although not historically considered an eosinophilic disease, some patients with COPD have evidence of eosinophil-associated inflammation.^123^ In the phase 3 GALATHEA and TERRANOVA studies of benralizumab in patients with moderate-to-very severe COPD, benralizumab did not reduce COPD exacerbations compared with placebo.^124^ Analysis of baseline characteristics identified those with elevated bEOS, ≥3 exacerbations in the past year and receiving triple therapy (ICS/LABA/LAMA) are most likely to benefit from treatment with benralizumab.^125^ A *post hoc* analysis of the two studies showed that, in the responder population, benralizumab 100 mg reduced risk of recurrent COPD exacerbations in 30- and 90-day periods after an initial exacerbation compared with placebo.^126^ The ongoing phase 3 RESOLUTE study will evaluate efficacy and safety of benralizumab 100 mg in patients with moderate-to-very severe COPD, ≥2 moderate/severe COPD exacerbations in the past year despite triple therapy and bEOS ≥300/μL.^127^

#### Potential future directions in eosinophil-associated diseases

Treatment with OCS is known to decrease bEOS, with real-world data suggesting reductions of 34% and 36% in patients with baseline bEOS ≥300/μL or ≥400/μL, respectively.^128^ Thus, the use of OCS in many patients may mask eosinophilia, potentially leading to physicians not considering and under-utilising anti-IL-5/Rα treatments, such as benralizumab. Although the PONENTE study assessed tapering OCS doses on benralizumab treatment,^69^ studies and guidance on how and whether to taper OCS dose prior to initiation of benralizumab treatment are limited. Indeed, real-world data suggest that the effect of OCS on bEOS may take several weeks to return to baseline upon discontinuation.^128^ Further studies are required to: (1) better understand if a lower bEOS cut-off is required for the use of anti-IL-5/Rα treatments in patients receiving OCS, and (2) provide physicians with guidance on whether and how to taper or discontinue OCS usage prior to testing for elevated bEOS and initiating anti-IL-5/Rα treatments.

Besides the known role of eosinophils in several lung diseases, eosinophilia (in bronchoalveolar lavage fluid) has also been associated with increased risk of chronic lung allograft dysfunction (CLAD) following lung transplantation.^129^ It remains to be determined if modulation of eosinophils through therapeutic intervention would prevent or treat CLAD.

Direct comparisons of the efficacy and safety of benralizumab with biologics other than mepolizumab are limited, particularly in eosinophilic conditions other than severe asthma. Additional studies or analyses are warranted to help guide physicians in selecting the most appropriate treatment for their patients.

Eosinophilia has been implicated in cutaneous AEs following cancer treatment.^130^ A single-arm, phase 2 study showed treatment with benralizumab can ameliorate eosinophil-related cutaneous AEs associated with systemic cancer therapies, particularly alpelisib and enfortumab vedotin; further investigation in placebo-controlled trials is warranted.^131^

## Conclusion

A wealth of clinical study data and real-world evidence supports the safety and efficacy of benralizumab for treatment of SEA. Benralizumab has also been approved in some countries for the treatment of EGPA. Through its ability to remove eosinophils in multiple tissues, benralizumab has potential to be a treatment option for a wide range of eosinophil-associated diseases, including HES, acute eosinophilic exacerbations, and COPD; however, results are awaited, or further studies are needed. Benralizumab may also be a useful tool for dissecting the role of eosinophils in inflammatory diseases.

## Abbreviations

ACQ-6, Asthma Control Questionnaire-6; AD, atopic dermatitis; ADCC, antibody-dependent cellular cytotoxicity; AE, adverse event; AER, annual exacerbation rate; AIC, allergen inhalation challenge; BD, bronchodilator; bEOS, blood eosinophils; CI, confidence interval; CLAD, chronic lung allograft dysfunction; COPD, chronic obstructive pulmonary disease; CRSwNP, chronic rhinosinusitis with nasal polyps; CSU, chronic spontaneous urticaria; CT, computed tomography; EGPA, eosinophilic granulomatosis with polyangiitis; EoE, eosinophilic esophagitis; EOT, end of treatment; FAO, fixed airflow obstruction; FeNO, fractional exhaled nitric oxide; FEV_1_, forced expiratory volume in 1 second; FVC, forced vital capacity; GINA, Global Initiative for Asthma; HCRU, healthcare resource utilisation; HES, hypereosinophilic syndrome; ICS, inhaled corticosteroid; Ig, immunoglobulin; IL, interleukin; IL-5R, interleukin-5 receptor; LABA, long-acting beta agonist; LAMA, long-acting muscarinic antagonist; LAR, late asthmatic response; LS, least-squares; mAb, monoclonal antibody; MRI, magnetic resonance imaging; NK, natural killer; NP, nasal polyps; OCS, oral corticosteroids; OLE, open-label extension; PD, pharmacodynamics; PK, pharmacokinetics; Q4W, once every 4 weeks; Q8W, once every 8 weeks; SAE, serious adverse event; SC, subcutaneous; SEA, severe eosinophilic asthma; SGRQ, St George's Respiratory Questionnaire; SNOT-22, Sino-Nasal Outcome Test-22; TNF, tumour necrosis factor; TNFR1, tumour necrosis factor receptor 1; URTI, upper respiratory tract infection; US, United States; VDP, ventilation-defect-percentage.

## Authors contributions

All authors contributed equally to the writing, reviewing and editing of this manuscript, and agreed to submission of the final draft.

## Submission declaration

All the authors have reviewed the manuscript and agreed to submit it by acknowledging the submission declaration.

## Disclosure on use of generative artificial intelligence (AI) and AI-assisted technologies

Nothing to disclose.

## Funding source

The development of this review was funded by 10.13039/100004325AstraZeneca.

## Declaration of competing interest

Stephanie Korn declares grants/funds: AstraZeneca, GlaxoSmithKline, Novartis, Sanofi-Genzyme, Teva; personal fees for lectures and advisory boards: AstraZeneca, GlaxoSmithKline, Novartis, Sanofi-Genzyme, Teva. Eugene R. Bleecker declares contracts for clinical trials: AstraZeneca, Sanofi-Genzyme; consulting fees: AstraZeneca, GlaxoSmithKline, Sanofi-Genzyme. Arnaud Bourdin declares grants/funds: Boehringer Ingelheim; personal fees: AstraZeneca, Boehringer Ingelheim, Chiesi, GlaxoSmithKline, Novartis, Sanofi-Regeneron; clinical trial investigator: Acceleron, Actelion, Galapagos, Merck Sharpe & Dohme, Nuvaira, Pulmonx, United Therapeutic, Celltrion, Vertex. Christopher McCrae declares past employment: AstraZeneca; and may own employee stocks and/or stock options: AstraZeneca. Maria L. Jison declares employment: AstraZeneca; employee stocks and/or stock options: AstraZeneca. Andrew Menzies-Gow declares employment: AstraZeneca; employee stocks and/or stock options: AstraZeneca; attendance at advisory boards: AstraZeneca, GlaxoSmithKline, Novartis, Sanofi, Teva; speaker fees: AstraZeneca, Novartis, Sanofi, Teva; participation in research with AstraZeneca for which his institution has been remunerated; consultancy agreements: AstraZeneca.
